# Use of relevancy and complementary information for discriminatory gene selection from high-dimensional gene expression data

**DOI:** 10.1371/journal.pone.0230164

**Published:** 2021-10-06

**Authors:** Md Nazmul Haque, Sadia Sharmin, Amin Ahsan Ali, Abu Ashfaqur Sajib, Mohammad Shoyaib

**Affiliations:** 1 Institute of Information Technology, University of Dhaka, Dhaka, Bangladesh; 2 Department of Computer Science & Engineering, Islamic University of Technology, Dhaka, Bangladesh; 3 Department of Computer Science & Engineering, Independent University, Dhaka, Bangladesh; 4 Department of Genetic Engineering & Biotechnology, University of Dhaka, Dhaka, Bangladesh; Chuo University, JAPAN

## Abstract

With the advent of high-throughput technologies, life sciences are generating a huge amount of varied biomolecular data. Global gene expression profiles provide a snapshot of all the genes that are transcribed in a cell or in a tissue under a particular condition. The high-dimensionality of such gene expression data (*i*.*e*., very large number of features/genes analyzed with relatively much less number of samples) makes it difficult to identify the key genes (biomarkers) that are truly attributing to a particular phenotype or condition, (such as cancer), *de novo*. For identifying the key genes from gene expression data, among the existing literature, mutual information (MI) is one of the most successful criteria. However, the correction of MI for finite sample is not taken into account in this regard. It is also important to incorporate dynamic discretization of genes for more relevant gene selection, although this is not considered in the available methods. Besides, it is usually suggested in current studies to remove redundant genes which is particularly inappropriate for biological data, as a group of genes may connect to each other for downstreaming proteins. Thus, despite being redundant, it is needed to add the genes which provide additional useful information for the disease. Addressing these issues, we proposed Mutual information based Gene Selection method (*MGS*) for selecting informative genes. Moreover, to rank these selected genes, we extended *MGS* and propose two ranking methods on the selected genes, such as *MGS*_*f*_—based on frequency and *MGS*_*rf*_—based on Random Forest. The proposed method not only obtained better classification rates on gene expression datasets derived from different gene expression studies compared to recently reported methods but also detected the key genes relevant to pathways with a causal relationship to the disease, which indicate that it will also able to find the responsible genes for an unknown disease data.

## Introduction

Genes are the physical and functional units of hereditary genetic information. The activity and/or expression level of a gene affects the synthesis of downstream protein(s) that dictates specific functionality in a cell. Therefore, the properties as well as the expression levels of a particular set of genes are responsible for a particular phenotype such as disease or tissue morphology. Those genes which are able to differentiate between different states (such as normal vs. diseased, quiescent vs. proliferating, adult vs. stem cells, etc.) of cells are called informative genes or biomarkers (a measurable indicator of a particular state). Identification of these informative genes is very important for elucidating developmental and disease mechanisms, disease diagnosis, drug development, etc. Especially, for the identification of different cancers, these informative genes may provide invaluable information for the improvement of diagnosis, prognosis, and treatment. For a set of known diseases, such informative genes are already identified using wet-lab verification. A computational method that can identify these known informative genes can be considered as a reliable method. Again, for known diseases, there might be few more informative genes (due to ethnicity variation) which are responsible for that disease. More importantly, for a new disease, these informative genes are unknown. Identifying these genes through wet-lab techniques are costly and time consuming. These time and cost can be significantly reduced by a reliable computation based method which is the main objective of this paper.

Usually, studies to generate disease specific gene expression profiles such as cancer comprise of a small number of control and patient samples, but tens of thousands of genes (high dimensional data) in each sample where only a few of the genes are responsible for a disease. Identification of a small subset of differentially expressed genes among thousands in cancerous cells compared to the normal ones is a challenging task and considered as NP (non-deterministic polynomial time) hard or NP-complete [1]. Therefore, the feature/gene selection methods can be a convenient and useful way to find a subset of genes relevant to a particular cancer. In this paper, we use the terms “gene” and “feature” interchangeably.

Till to date, several gene selection methods have been proposed, particularly for cancer data classification [2–4]. These methods can be categorized into three types, such as “Filter”, “Wrapper”and “Hybrid” [5]. Among them, filter based methods are more popular as these can assess the property of features without being dependent on any particular classifier. Filter based methods select a subset of features based on some criteria such as correlation coefficient [6], t-statistics [7], distance [8, 9], Mutual Information(MI) [10–13]. Among these, MI based methods are popular for feature selection due to their ability to capture non-linear dependencies between features. One of the recent works used Minimum Redundancy Maximum Relevance (MRMR) [3] where each gene was selected incrementally to hold the highest discriminatory power (relevancy) with the target class (control/cancer) and the lowest dependency (redundancy) with other selected genes. However, in this method, bias corrections (errors occurred due to finite number of samples) are not considered and there are some genes which add some additional information about the class that are discarded. To solve this issue, a new information theoretic measure such as complementary (additional) information that a gene has about the class (which is not found in the already selected subset of genes) has been proposed in [11, 14]. These methods attempted to estimate the joint mutual information of a feature subset with the class. Another method, modified Discretization and feature Selection based on Mutual information (mDSM) [11] includes bias correction and captures complementary information. Relaxmrmr [14] and DSbM [13] add a higher order term, namely feature-feature interaction in addition to the complementary information. However, all these methods discard those genes considering as redundant which may provide complementary information about a particular disease (class). The exclusion of a gene considering only a pair-wise correlation may hamper of finding informative and distinguishable genes because a group of genes is connected to each other to perform a particular function.

In contrast to filter based methods, wrapper based methods are classifier dependent. Wrapper based methods select the most discriminant subset of features by minimizing the prediction error of a particular classifier [15]. Support Vector Machine based on the Recursive Feature Elimination (SVM-RFE) [2] is considered to be one of the best performing wrapper methods. It ranks the genes using SVM and selects the important genes using recursive feature elimination strategy. Different variants of SVM-RFE have also been proposed [16, 17]. Although the wrapper based feature selection methods provide better performances, these methods become computationally expensive when the feature size grows. Moreover, these methods may not provide the optimal solution for other classifiers [18].

To combine the advantages of wrapper and filter based methods, a hybrid approach was introduced which first selected candidate gene subset from the original gene set via computationally-efficient filter method and then the candidate gene subset was further refined by wrapper method. An example of a hybrid method named Information Guided Interactive Search (IGIS) [19] that selected the best set of genes based joint MI. However, this method selected more genes than the wrapper or the hybrid algorithms. Addressing the limitations of IGIS, improved Interaction information-Guided Incremental Selection (*IGIS*+) [20] was proposed, where the first gene was selected based on the highest accuracy using KNN and CART classifiers and utilized Cohen’s *d* test to add a new gene into the selected gene subset. One major limitation of *IGIS*+ is that it uses several handcrafted thresholds. There are several popular bio-inspired algorithms to find out the optimal set of features. Almugren et al. in [21] provided an extensive review of the bio-inspired hybrid methods. Alshamlan et. al. proposed a hybrid artificial bee colony [22] and a genetic bee colony [23] optimization method that uses MRMR criterion. El Akadi et al. [24] proposed a genetic algorithm based on MRMR criterion. In these methods, MRMR criterion is used to filter noise and redundant genes in the high-dimensional microarray data and then the bio-inspired algorithm uses the classifier accuracy as a fitness function to select the highly discriminating genes. Particle swarm optimization is a kind of bio-inspired swarm intelligence optimization method which was used to select informative genes [25]. In this method, informative genes are selected in autism spectrum disorder by utilizing a combination of various statistical filters and a wrapper-based Geometric Binary Particle Swarm Optimization-Support Vector Machine (GBPSO-SVM) algorithm. Another recent hybrid method was introduced by Hameed et al. [26] named HDG-select. It provides a graphical user interface that uses mixed filter-GBPSO-SVM for feature selection, while SVM is used for disease classification. Most bio-inspired algorithms use local searches with random restart or population based methods. However, these algorithms still can get stuck at a local optimum. In order to solve the optimization problems globally, a parallel search strategy was attempted in [27]. It incorporated parallel search strategies based on semi-definite programming or quadratic programming that can find the feature subset in polynomial time.

Recently, deep learning based methods show better accuracy in different classification problems such as image [28, 29], text [30] or audio [31] classification. Deep learning based methods have also been proposed for gene expression data [4] where the authors developed a new model namely Forest Deep Neural Network (*fDNN*) that incorporated deep neural network (DNN) with random forest (RF) to solve the problem of learning from small sample data having a large number of genes. RF was used to reduce the dimension of these datasets by detecting the important genes in a supervised manner. This new feature representation was then fed into DNN to predict the outcomes. However, this method does not make use of the main advantage of deep learning, which is automatic feature extraction in solving classification problems. On the other hand, using a neural network as a black box to extract new features from gene expression data reduces the interpretability of the classifier, which is important in studies such as disease (cancer) classification.

We in this paper choose filter based methods for gene selection instead of deep learning or wrapper/hybrid methods due to the useful properties that filter based methods have, namely interpretability, classifier independence, and superior performance. Moreover, we adopt filter based methods that use selection criteria based on MI for reasons mentioned previously. However, one of the challenges of MI based methods is to reliably estimate the *MI* when the dataset is high-dimensional but contains few samples. Gene expression datasets have this characteristic. There has been a lot of effort to better approximate the *MI*. Among them, one of the recent works is modified Discretization and Selection of feature based on *MI* (*mDSM*) [11], where the authors showed that during the calculation of MI for finite samples, there exist some errors (bias) for all the three terms namely relevancy, redundancy and complementary information. Moreover, for selecting a feature, they proposed to use *χ*^2^ statistics by showing that these terms follow *χ*^2^ distribution. Despite having a few good characteristics, MI based methods might discard informative genes by incorporating the term *redundancy* in gene expression data [20]. Note that, usually most of the existing works improve the classification accuracy whereas it is also important to identify genes that improve classification accuracy and are relevant to a particular disease [5].

To solve the aforementioned problems, we propose a new MI based filter method, namely Mutual information based Gene Selection (*MGS*) that achieves better classification performance as well as captures biological significance with high dimensional data. The main contributions of this study are as follows: first, a gene selection technique is proposed for identifying the discriminating genes relevant to a particular disease based on their relevancy and complementary information. Second, a statistical test is used to select genes without a handcrafted threshold. Third, two ranking techniques are proposed to rank the selected genes and select top *η* genes as biomarkers for disease classification. Finally, the selected informative genes are validated using already wet-lab tested results with a causal relationship to a particular type of disease (phenotype) with the hope that this method will also work well for unknown disease data.

## Materials and methods

### Dataset description

To find the informative genes and to assess the performance of the proposed method compared to the existing ones, we choose datasets that have different characteristics, such as balanced and imbalanced datasets, and small and relatively large samples having different diseases. We used seven different gene expression datasets such as GDS3341 [32], GDS3610 [33], GDS4824 [34], GSE106291 [35], GDS4431 [36], GDS5306 [37] and GDS6063 [38] retrieved from the Gene Expression Omnibus (GEO) database [39] at the National Center for Biotechnology Information (https://www.ncbi.nlm.nih.gov). These datasets are grouped into two sets based on the distribution of control and disease samples: Balanced datasets (GDS3341, GDS3610, GDS4824 and GSE106291) and Imbalanced datasets (GDS4431, GDS5306 and GDS6063) having small and relatively large samples. The description of datasets is given in Table 1. Expression data of multiple probes for the same gene were merged. At first, we download these datasets (.soft extension) from the NCBI site, and it is transformed into CSV format taking samples and features (genes). Here, features are defined by probe id and gene symbol. There are various cases where probe id is different but same gene symbol name. In this case, we merge these gene symbols by taking their mean. In addition, the genes which have no expression values over the samples are discarded. All these datasets contained much less number of samples compared to the number of genes. These datasets are publicly available at https://doi.org/10.6084/m9.figshare.16680355.

**Table 1:** Summary of the datasets used in this study.

Dataset type	Sample size	Dataset ID	Description	Total samples	Control samples	Disease samples	Features/genes
Balanced	Small	GDS6063	Influenza A infected plasma-cytoid dendritic cells(pDC)	10	5	5	36825
		GDS5306	Breast cancer brain metastasis specimens and non-metastatic primary breast tumors	38	19	19	32389
	Relatively large	GDS4431	Peripheral blood lymphocytes of autistic and non-autistic child	146	69	77	30803
Imbalanced	Small	GDS4824	Prostate cancer	21	8	13	30872
		GDS3610	Nasopharyngeal carcinoma	28	3	25	14126
		GDS3341	Nasopharyngeal carcinoma	41	10	31	30865
	Relatively large	GSE106291	Acute myeloid leukemia	235	71	164	21403

### Gene selection and validation processes

**Fig 1:**
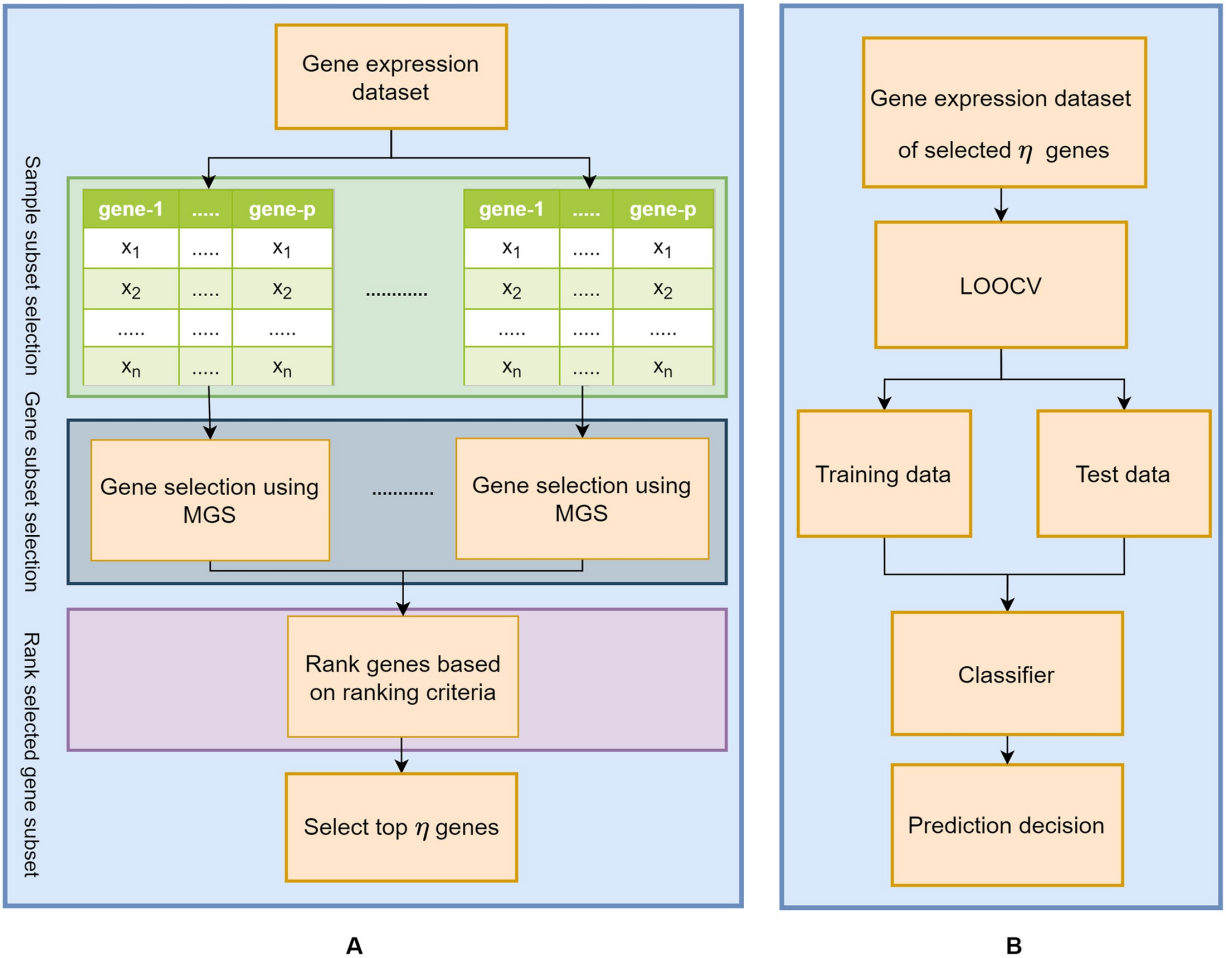
Overall process of the proposed method. (A) Gene selection. (B) Classification.

The overall process of the proposed MI based Gene Selection (*MGS*) is shown in Fig 1. We first identified the informative genes using *MGS* and then selected top *η* genes by ranking them according to their performance (Fig 1A). Finally, we use these *η* genes for classification (Fig 1B) and validating the biological significance. The following subsections describe our method with further details.

### Gene selection

For the identification of a gene subset, we used a filter based gene selection method that approximated the joint *MI* with respect to the class variable. In order to identify an informative gene subset, we first subdivided the given gene expression dataset into *K* subsets and applied K-fold cross validation (KFCV). However, when the number of samples (*n*) is small (*n* < 100), Leave One Out Cross Validation (LOOCV) is applied where *K* = 1. In *MGS*, we incorporated a variant of the *mDSM* [11] by modifying the selection criteria so that it can identify biologically relevant genes for a disease. The accumulation of all genes identified by *MGS* from *K* different subsets was defined here as selected gene subset (*G*_*S*_). Finally, to rank these selected gene subset (*G*_*S*_), two ranking criteria namely *MGS* frequency-based ranking (*MGS*_*f*_) and *MGS* Random Forest (RF) based ranking (*MGS*_*rf*_) were proposed to select the top *η* genes as biomarkers.

- **Gene subset (*G*_*S*_) selection**: To measure how much information a particular gene expression dataset provided for the identification of a disease, we calculated MI between the expression values of a gene *g*_*i*_ and the class variable *C*. This MI represented the relevancy of a gene that revealed the degree of importance of that gene in disease data classification. Note that, before calculating the MI, the gene expression data was discretized which was necessary for noise reduction and data simplification, and thus resulted in maximizing the relevancy of a gene to the target class *C*. For calculating the relevance between *g*_*i*_ and *C*, MI was calculated using Eq 1.
  $$\begin{array}{c} J_{rel}\left(g_{i}\right)=I\left(g_{i}^{d_{i}};C\right)-\frac{\left(I-1)(K-1\right)}{2N\;\text{ln}\;2} \end{array}$$ (1)
  where, $g_{i}^{d_{i}}$ denotes gene *g*_*i*_ with *d*_*i*_ discretization levels. The second term of the right hand side of Eq (1) is the bias correction term for calculating the relevancy where I, K and *N* represent the discretization levels of gene *g*_*i*_, the total number of classes in *C* and the total number of samples respectively. For each gene *g*_*i*_, the minimum discretization levels *d*_*i*_ was chosen for which *J*_*rel*_(*g*_*i*_) was greater than its *χ*^2^ critical value ($x_{C}^{2}\left(rel\right)$) and thus helped to determine whether the gene was significantly relevant or not. This test could be done as it could be shown that the relevancy followed *χ*^2^ distribution with (I−1)(K−1) degrees of freedom. The genes which satisfied the *χ*^2^ critical value were included in the candidate gene subset, *G*_*c*_. Then, these candidate genes, *G*_*c*_ were ranked in descending order based on the relevancy. As the top ranked gene was considered to be the most important, we included it to the selected gene subset *G*_*S*_ at first. Now, the second ranked one was evaluated for selection based on its score calculated using Eq 2.
  $$\begin{array}{cc} J_{MGS}\left(g_{i}\right)= & I\left(g_{i}^{d_{j}};C\right)-\frac{\left(I-1)(K-1\right)}{2N\;\text{ln}\;2}\\ & +\frac{1}{|G_{S}|}\sum_{g_{s}\in G_{S}}[I\left(g_{i}^{d_{j}};g_{s}|C\right)-\frac{(I-1)(J-1)K}{2N\;\text{ln}\;2}] \end{array}$$ (2)
  Here, along with relevancy, the complementary information ($I(g_{i}^{d_{j}};g_{s}|C)$) of a new gene was also calculated. The complementary information $I(g_{i}^{d_{j}};g_{s}|C)$ due to *g*_*i*_ for the already selected gene in *g*_*s*_ revealed the dependency among those genes while identifying the class variable *C*. Here, J represents the discretization levels of a gene in *G*_*S*_. The last term in Eq 2 is the bias correction for complementary information. While calculating the value of *J*_*MGS*_, the discretization level (*d*_*i*_) of the *g*_*i*_ which was fixed using Eq (1) was also shifted by a small amount (±*δ*) to check whether the value of *J*_*MGS*_ is increasing because a small shifting of discretization might increase the value of *J*_*MGS*_ and this new discretization value was chosen dynamically considering the dependency among the genes. Now, for a particular gene (*g*_*i*_), if the value of *J*_*MGS*_ was larger than the *χ*^2^ critical value ($\chi_{C}^{2}\left(MGS\right)$), then it was placed into the selected gene subset. When the relevancy and complementary information of a *g*_*i*_ was significant, it was selected, otherwise discarded. So, identification of genes that maximize *J*_*MGS*_ indicated the genes which were strongly relevant with the class *C* with greater additional information would be adopted to the selected subset throughout this process.
  It is noteworthy to mention that a group of genes with similar expression values may exist which will be identified as redundant. However, if these have complementary (additional) information about the class, it is necessary to incorporate that gene into the selected subset even though these are redundant. Inclusion of the redundant genes is sensible because; usually a set of genes contributes mutually for a particular task in our body and these genes may share a similar or correlated expression profile. The biological importance of such inclusions is presented in the Results and discussion section. The whole procedure of selecting gene subset are illustrated in Algorithm **1**.
- **Rank the selected gene subset**: The same subset of genes was not always selected during the selection of genes by *MGS* at each iteration of LOOCV. For example, in a dataset having *n* number of samples, we used (*n* − 1) samples for training and the *n*^*th*^ sample for testing. After passing the training data to *MGS*, we got an informative selected gene subset. This was repeated *n* times and aggregated all the selected gene subsets (*G*_*S*_) and considered the union of these subsets to get *G*_*SU*_. Afterward, these genes in *G*_*SU*_ were ranked using one of the following two ranking criteria.
  - ***MGS*_*f*_**: This ranking was performed based on the following assumption. *Assumption*: The genes which are selected in every iterations are likely to have more discriminating power and biological significance.
    To quantify the *Assumption*, we computed the relative frequency of every selected gene, *S*_*i*_ in *G*_*SU*_ using Eq 3.
    $$\begin{array}{c} P\left(S_{i}\right)=\frac{F_{S_{i}}}{N_{G_{SU}}} \end{array}$$ (3)
    Here, $N_{G_{SU}}$, $F_{S_{i}}$ and *P*(*S*_*i*_) are the total number of genes in *G*_*SU*_, frequency of the selected gene *S*_*i*_ and the relative frequency of gene *S*_*i*_ respectively. For example, we had two selected gene subsets, *L*_1_ = {*g*_1_, *g*_3_, *g*_4_, *g*_5_, *g*_6_} and *L*_2_ = {*g*_1_, *g*_2_, *g*_4_, *g*_6_}. Here, the unique genes were *G*_*SU*_ = {*g*_1_, *g*_2_, *g*_3_, *g*_4_, *g*_5_, *g*_6_} and the frequencies of these unique genes were *F* = 2, 1, 1, 2, 1, 2 respectively. So, the relative frequencies were *P*(*S*_*i*_) = 2/6, 1/6, 1/6, 2/6, 1/6, 2/6. Thus, based on the *P*(*S*_*i*_), ranked genes were *G*_*SR*_ = *g*_1_, *g*_4_, *g*_6_, *g*_2_, *g*_3_, *g*_5_.
  - ***MGS*_*rf*_**: Informative genes have the ability to split the control and disease samples into two groups. To find the more informative genes, it is needed to rank the selected gene subset. In order to rank the genes *G*_*SU*_, it is necessary to measure how much information a gene contains. To measure the information content of a gene, we can use Information Gain (IG) criterion. IG is used in decision trees [40] to select features that reduces the entropy of the data most by splitting data into two groups (called the the left and right child in a decision tree). We used weighted IG given in Eq 4.
    $$\begin{array}{c} IG=\frac{N_{t}}{N}[H\left(node_{Parent}\right)-\frac{N_{L}}{N_{t}}*H\left(node_{Leftchild}\right)-\frac{N_{R}}{N_{t}}*H\left(node_{Rightchild}\right)] \end{array}$$ (4)
    Where, *N*_*t*_ is the number of samples at the current (parent) node, *N* is the total number of samples, *N*_*L*_ is the number of samples in the left child, and *N*_*R*_ is the number of samples in the right child. *H*(*node*) is the entropy at the node. The entropy was calculated using Eq 5.
    $$\begin{array}{c} H\left(node\right)=-\sum_{i=1}^{C}P_{i}logP_{i} \end{array}$$ (5)
    Here, *P*_*i*_ is the probability of the outcome/class, *i*. Each node in a DT contains a gene with its corresponding weighted IG. Besides, to make the weighted IG more robust, we used *M* number of DTs to construct a Random Forest and took the average of IGs for each gene *g*_*j*_ ∈ *G*_*SU*_ using Eq 6.
    $$\begin{array}{c} IG_{g_{j}}=\frac{1}{\sum_{i=1}^{V}\delta \left(v_{i}.g,g_{j}\right)}[\sum_{i=1}^{V}\delta \left(v_{i}.g,g_{j}\right)*v_{i}.IG] \end{array}$$ (6)
    Here, *V* = {*v*_*i*_, *v*_*i*+1_,‥,*v*_*k*_} = {(*g*_*i*_, *IG*_*i*_), (*g*_*i*+1_, *IG*_*i*+1_),‥,(*g*_*k*_, *IG*_*k*_)} and *k* is the total number of nodes in the random forest. That is, for each node of the random forest, we stored the corresponding gene and its weighted IG in *V*. *δ*(*v*_*i*_.*g*, *g*_*j*_) = 1 if *v*_*i*_.*g* = *g*_*j*_, and 0, otherwise.
    This average score can be used as the importance score of each gene. In our case, this importance score represented how important a particular gene was to explain the target class. Then, based on the importance score, the genes from *G*_*SU*_ were ranked in descending order. And finally, from the ranked genes, top *η* genes were taken as biomarkers and the performance metrics were calculated.

**Algorithm 1**: **MGS**

**Input**: Set of genes *G*, maximum discretization level *max*_*d*_

**Output**: Selected subset of genes, *G*_*S*_

1: Initialize candidate gene subset (*G*_*c*_), its discretization level (*D*_*c*_) and relevance (*J*_*c*_) with ∅.

2: **for** each *g*_*i*_ ∈ *G* **do**

3:  **for all** *j* = 2 to *max*_*d*_ **do**

4:   Discretize *g*_*i*_ with *j* intervals

5:   Calculate *J*_*rel*_(*g*_*i*_) using Eq (1)

6:   **if** $J_{rel}\left(g_{i}\right)>\chi_{C}^{2}\left(rel\right)$ **then**

7:    *D*_*c*_ ⇐ *D*_*c*_ ∪ *j*;

8:    *J*_*c*_ ⇐ *J*_*c*_ ∪ *J*_*rel*_(*g*_*i*_)

9:    *G*_*c*_ ⇐ *G*_*c*_ ∪ *g*_*i*_

10:    **break**

11:   **end if**

12:  **end for**

13: **end for**

14: Sort *G*_*c*_ in decreasing order based on their corresponding *J*_*c*_ values

15: Select *g*_1_ and the corresponding *d*_1_ from *G*_*c*_ with max *J*_*c*_

16: *G*_*S*_ ⇐ {*g*_1_}

17: *D*_*S*_ ⇐ *d*_1_

18: *G*_*c*_ ⇐ *G*_*c*_ \ *g*_1_

19: **for** each *g*_*i*_ ∈ *G*_*c*_, *d*_*i*_ ∈ *D*_*c*_, *j*_*i*_ ∈ *J*_*c*_ **do**

20:  Initialize threshold, *T* ⇐ 0

21:  **for all** *j* = *d*_*i*_ − *δ* to *d*_*i*_ + *δ* **do**

22:   Discretize *g*_*i*_ with *j* intervals

23:   Calculate *J*_*MGS*_(*g*_*i*_) using Eq (2)

24:   **if** $J_{MGS}\left(g_{i}\right)>\chi_{C}^{2}\left(MGS\right)$ **then**

25:    *d*_*i*_ ⇐ *j*;

26:    $T⇐\chi_{C}^{2}\left(MGS\right)$

27:    *j*_*i*_ ⇐ *J*_*MGS*_(*g*_*i*_)

28:   **end if**

29:  **end for**

30:  **if** *j*_*i*_ > *T* **then**

31:   *G*_*S*_ ⇐ *G*_*S*_ ∪ *g*_*i*_

32:   *D*_*S*_ ⇐ *D*_*S*_ ∪ *d*_*i*_

33:  **end if**

34:  *G*_*c*_ ⇐ *G*_*c*_ \ *g*_*i*_

35: **end for**

36: **Return** *G*_*S*_

### Classification

For classification, as shown in Fig 1B, only the selected top *η* genes from the previous step were used in the train and test data to predict the outcome. To assess the performance of a gene selection method, we considered two performance metrics, *accuracy* and Area Under the Receiver Operating Characteristic Curve (*AUROC*). *Accuracy* is the percentage of samples that are predicted as the true class. *AUROC* represents degree or measure of separability between classes, and it can be used both balanced and imbalanced datasets, specially imbalanced dataset. *ROC* is a probability curve of a classifier at various thresholds. It plots a curve based on the true positive rate (TPR) and false positive rate (FPR) represented in Eqs 7 and 8.

$$\begin{array}{c} TPR=\frac{TP}{TP+FN} \end{array}$$ (7)

$$\begin{array}{c} FPR=\frac{FP}{FP+TN} \end{array}$$ (8)

here, “TP” and “TN” are the numbers of positive and negative samples that are correctly classified. “FP” is the number of negative-class samples misclassified as the positive class, and “FN” is the number of positive-class samples misclassified as the negative class. To compute the points in a *ROC* curve, *AUROC* computes an aggregate measure of various thresholds. For our experiments, the reported results were calculated by taking the average over the KFCV/LOOCV process for these two metrics. Selection of the informative features in the *MGS* step was implemented using MATLAB and ranking these informative features in *MGS*_*f*_ and *MGS*_*rf*_ step was implemented using Python with the package scikit-learn [41]. To evaluate the performance of the proposed and existing methods, different classifiers such as SVM, RF classifiers, XGboost [42], PE*k*NN [43] can be used. In this paper, we only use two simple classifiers namely SVM (linear kernel) and Random Forest to compare different methods. These classifiers were implemented using Python with Scikit-learn packages. The source code is available, which can be downloaded from GitHub (https://github.com/Shisir/MGS). All experiments are conducted using a PC with Core-i7, CPU (3.60GHz x 8), and 16GB of RAM.

### Biological interpretation of the selected genes

We used NetworkAnalyst [44] to interpret the biological significance of the selected genes. NetworkAnalyst is a bioinformatics platform to interpret gene expression data within the context of protein-protein interaction (PPI) networks which is widely used in many renowned researches such as [45–47]. It uses well-established walktrap algorithm [48]. The general idea of walktrap algorithm is that if we perform random walks on the PPI network, the walks are more likely to stay within the same module (nodes those are closely connected to each other) because there are only a few edges that lead outside a given module. Then, to assess the goodness of these modules, modularity [49] is used. The modularity quality function is based on the comparison with a random graph that is not expected to have a cluster structure. As the input of NetworkAnalyst, we used top *η* selected genes for each dataset determined by our proposed and the previously described methods [4, 11, 20, 40]. Only the PPI networks that accommodate these genes with False Discovery Rate (FDR) < 0.05 were considered. FDR is more stringent than the p-value and has become invaluable in transcriptional profiling, and large-scale bioinformatics analysis in general. Since the nature of the biological samples in the datasets was known, we assessed the performance of the compared methods based on their abilities to identify the key pathways affected in the corresponding sample types.

### An illustrative example

Here, we present a toy example in order to demonstrate the overall mechanism of the proposed method. Let us assume a dataset having 20 genes (*g*_1_, *g*_2_, ‥, *g*_20_) with 10 samples where the distribution of control and disease samples are seven and three, respectively. First, in *MGS*, we calculate the relevance for the expression values of each gene with the minimum discretization level using Eq 1 and the first gene is selected which has the highest relevance. Then, the next gene subset is selected by a small change (±*δ*) of the initial discretization level of each gene to maximize the *J*_*MGS*_ criterion mentioned in Eq 2. In this way, the genes are assessed considering its interaction with other genes. In this example, 10 out of 20 genes are selected as a selected gene subset (*G*_*S*_). After that, to rank the selected gene subset, in *MGS*_*f*_, the relative frequencies of the selected genes are recorded using Eq 3 over the LOOCV. On the other hand, in *MGS*_*rf*_, an importance score is given to every candidate gene using Eqs 4–6 over the LOOCV. Table 2 represents the relative frequency distribution and importance score of the selected genes after applying *MGS*_*f*_ and *MGS*_*rf*_, respectively. From these ranking, top *η*(= 5) genes are considered as biomarkers. So, the top 5 ranked gene subset of *MGS*_*f*_ and *MGS*_*rf*_ are *g*_2_, *g*_3_, *g*_5_, *g*_9_, *g*_12_ and *g*_3_, *g*_2_, *g*_13_, *g*_9_, *g*_5_, respectively. Finally, using these biomarkers, two classifiers (*SVM* and *RF*) are applied to compute *accuracy* and *AUROC*. Besides, these biomarkers are also assessed for biological interpretation.

**Table 2:** Relative frequency distribution (*MGS*_*f*_) and importance score (*MGS*_*rf*_) of the selected gene subset.

Gene	*g*_2_	*g*_3_	*g*_5_	*g*_9_	*g*_12_	*g*_13_	*g*_15_	*g*_17_	*g*_19_	*g*_20_
*MGS*_*f*_ score	0.8	0.8	0.7	0.6	0.6	0.5	0.4	0.4	0.2	0.2
*MGS*_*rf*_ score	0.92	0.97	0.73	0.76	0.69	0.89	0.44	0.57	0.39	0.21

## Results and discussion

We compared the performances of our proposed filter based methods (*MGS*_*f*_ and *MGS*_*rf*_) to other already renowned methods such as *RF* (filter) [40], *fDNN* (embedded) [4], *IGIS*+ (hybrid) [20], *HDG* (hybrid) [26] and *mDSM* (filter) [11].

In this study, we applied the aforementioned methods on seven gene expression datasets. Here, we first discussed the classification performance of all methods using the top *η* genes and then compared the performance of all methods for different numbers of top *η* genes to assess the robustness of our method. In situations where the gene selection methods selected less than 10 genes, we used all the selected ones in further analysis. Finally, we provided a biological interpretation of the top ten (*η*) selected genes. For a fair comparison, we followed the same training and testing protocol for all the methods. With *RF*, *fDNN* and *MGS*_*rf*_ (where random forest was used), we applied 300 decision trees.

### Classification performance

Tables 3 and 4 summarized the comparative results of the proposed methods along with the existing methods on balanced and imbalanced datasets respectively and the values in boldface represent the classification performance of the best performing method for a particular classifier. For balanced datasets, the average *accuracy* and *AUROC* indicated the superiority of *MGS*_*f*_ and *MGS*_*rf*_ against other five gene selection methods on two different classifiers (Table 3). With GDS6063 and GDS5306 datasets, *MGS*_*f*_ and *MGS*_*rf*_ performed better than the other reported methods. Although *fDNN* performed slightly better than *MGS*_*f*_ and *MGS*_*rf*_ with GDS5306 dataset, the biological significance of the selected genes was not as satisfactory as our methods (discussed later).

**Table 3:** Classification accuracy and AUROC of different methods for balanced datasets.

Methods	Dataset: GDS6063				Dataset: GDS5306				Dataset: GDS4431			
	*Accuracy*		*AUROC*		*Accuracy*		*AUROC*		*Accuracy*		*AUROC*	
	SVM	RF	SVM	RF	SVM	RF	SVM	RF	SVM	RF	SVM	RF
*RF*	0.700	0.700	0.898	0.766	0.447	0.500	0.583	0.710	0.561	0.625	0.632	0.719
*fDNN*	0.900	0.800	0.900	0.932	**0.690**	**0.888**	0.814	0.926	0.697	0.742	0.750	0.824
*IGIS*+	0.600	0.400	0.460	0.480	0.605	0.868	0.610	0.890	0.616	0.705	0.560	0.780
*HDG*	0.900	0.891	0.953	0.972	0.685	0.763	0.798	0.916	0.725	0.740	0.787	0.788
*mDSM*_*f*_	0.900	0.900	0.920	0.940	0.650	0.775	0.758	0.866	0.705	0.767	0.830	0.839
*mDSM*_*rf*_	0.900	0.900	0.920	0.940	0.650	0.775	0.758	0.866	0.705	**0.767**	**0.830**	0.839
*MGS*_*f*_	0.900	0.900	0.960	0.990	0.658	0.868	0.820	0.940	0.733	0.753	0.820	0.850
*MGS*_*rf*_	**0.900**	**0.900**	**0.960**	**0.990**	0.658	0.868	**0.812**	**0.940**	**0.733**	0.753	0.820	**0.850**

**Table 4:** Classification accuracy and AUROC of different methods for imbalanced datasets.

Methods	Dataset: GDS3341				Dataset: GDS4824				Dataset: GDS3610				Dataset: GSE106291			
	*Accuracy*		*AUROC*		*Accuracy*		*AUROC*		*Accuracy*		*AUROC*		*Accuracy*		*AUROC*	
	SVM	RF	SVM	RF	SVM	RF	SVM	RF	SVM	RF	SVM	RF	SVM	RF	SVM	RF
RF	0.878	0.878	0.955	0.940	0.476	0.476	0.289	0.389	0.679	0.893	0.253	0.507	0.698	0.702	0.277	0.622
*fDNN*	1.00	1.00	1.00	1.00	0.952	1.00	1.00	1.00	0.750	0.893	0.560	0.827	0.766	**0.779**	0.778	0.783
*IGIS*+	0.976	1.00	1.00	1.00	1.00	1.00	1.00	1.00	0.893	0.893	0.853	0.940	0.732	0.762	0.695	0.765
*HDG*	1.00	1.00	1.00	1.00	0.952	1.00	0.960	1.00	0.964	0.964	1.00	0.980	0.698	0.690	0.600	0.567
*mDSM*_*f*_	1.00	1.00	1.00	1.00	1.00	1.00	1.00	1.00	0.964	0.929	1.00	0.980	0.728	0.694	0.638	0.629
*mDSM*_*rf*_	1.00	1.00	1.00	1.00	1.00	1.00	1.00	1.00	0.964	0.929	1.00	0.980	0.698	0.689	0.400	0.5419
*MGS*_*f*_	1.00	1.00	1.00	1.00	1.00	1.00	1.00	1.00	0.964	0.929	0.960	0.973	0.757	0.762	0.764	0.793
*MGS*_*rf*_	1.00	1.00	1.00	1.00	1.00	1.00	1.00	1.00	**1.00**	**0.964**	**1.00**	**0.987**	**0.770**	0.757	**0.787**	**0.796**

For imbalanced datasets, the similar superiority of the *MGS*_*f*_ and *MGS*_*rf*_ could be observed in most of the cases compared to the existing methods (Table 4), which indicated that the proposed methods selected more informative genes. With GDS3341 and GDS4824 datasets, all methods except *RF* were able to perfectly differentiate the control and disease samples for both *SVM* and *RF* classifiers. The small number of samples compared to a large number of genes might be the reason behind the relatively poor performance of *RF*. Even though the other methods performed well for selecting the distinguishable genes between the control and disease samples, all these genes were not biologically informative (discussed later). With the GDS3610 and GSE106291 datasets, *MGS*_*f*_ and *MGS*_*rf*_ methods achieved better performances compared to the other methods except one (fDNN using RF classier with GSE106291 dataset). Besides the superiority of *MGS* in terms of accuracy, it also performed significantly better in many cases in comparison to most of the methods.

It is observed from the Tables 3 and 4 that *mDSM*, *fDNN* and *HDG* performed reasonably and *MGS* outperformed most of its competitive methods. Even though *MGS*_*f*_ performed better in most of the cases compared to the existing methods, *MGS*_*rf*_ performs slightly better than *MGS*_*f*_. It is because of the selection of informative genes based on the RF classifier. As the number of samples is relatively small with respect to the number of genes, *MGS*_*f*_ could easily overfit training samples and thus performed poorly for unseen data. *MGS*_*rf*_ solved the overfitting problem by using *RF* classifier [40].

### Comparison of performances for different number of genes

**Fig 2:**
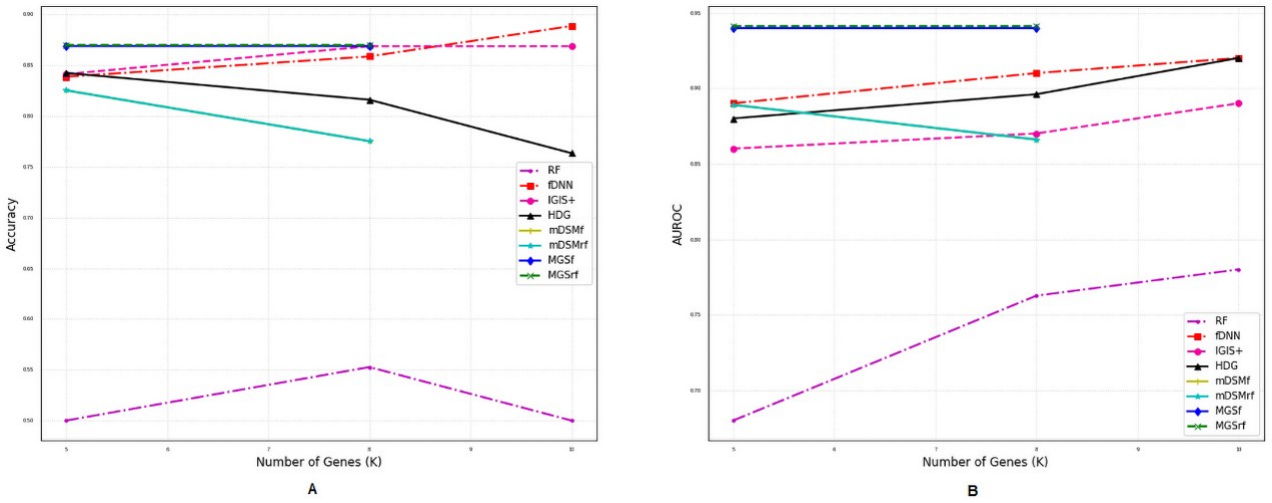
Performance comparison using different number of selected genes for the GDS5306 dataset. (A) Accuracy. (B) AUROC.

**Fig 3:**
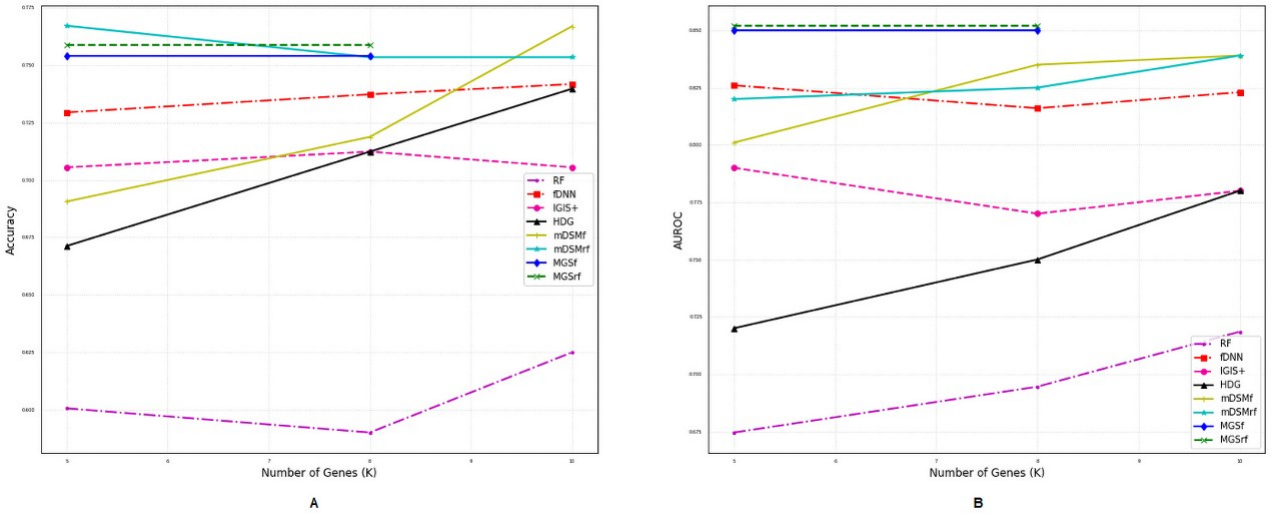
Performance comparison using different number of selected genes for the GDS4431 dataset. (A) Accuracy. (B) AUROC.

**Fig 4:**
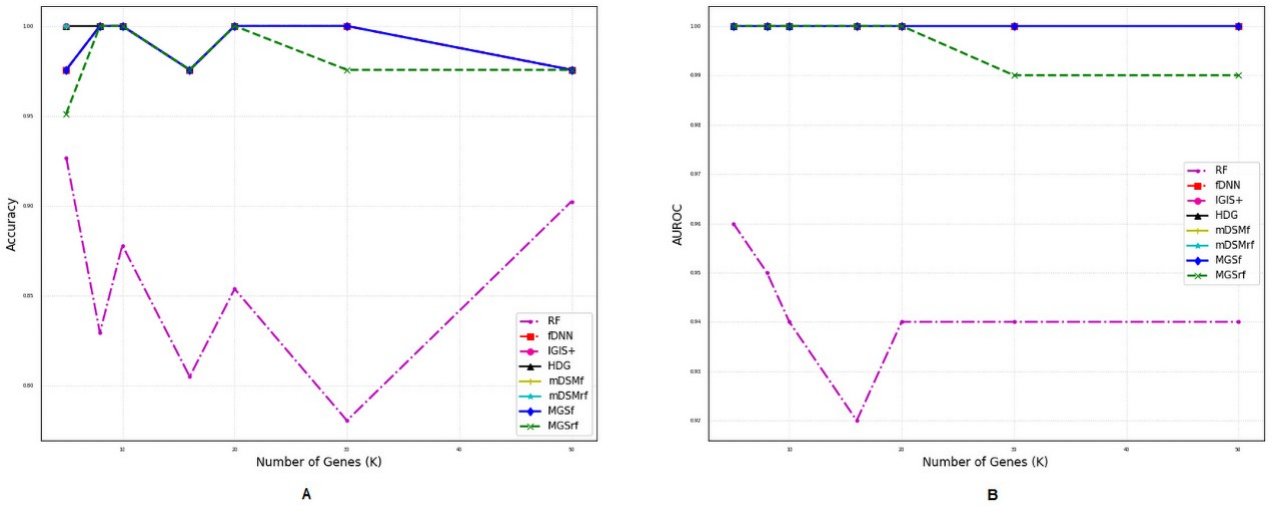
Performance comparison using different number of selected genes for the GDS3341 dataset. (A) Accuracy. (B) AUROC.

**Fig 5:**
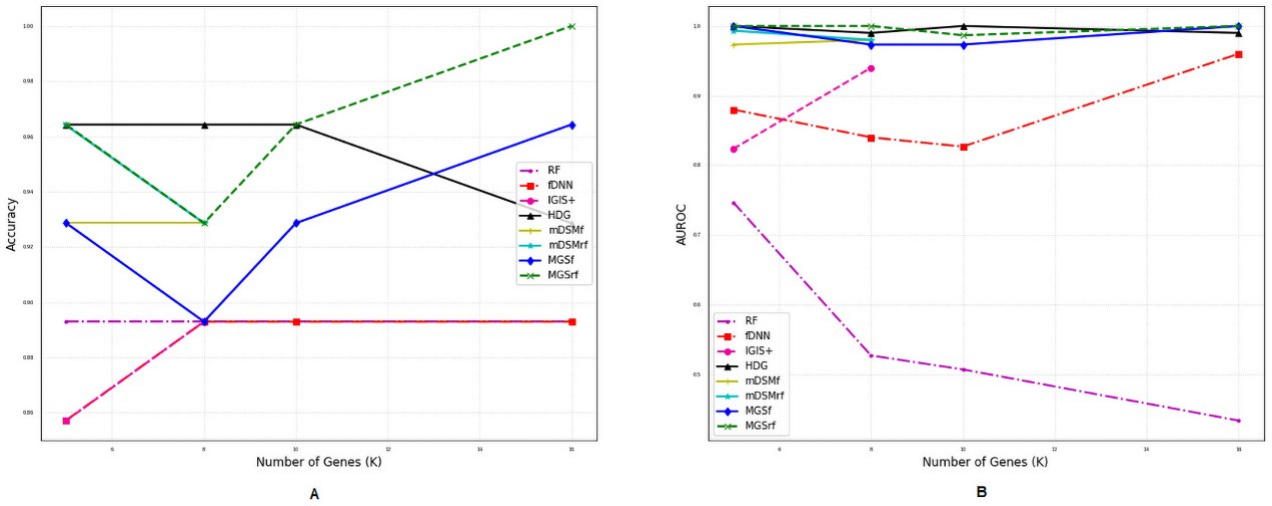
Performance comparison using different number of selected genes for the GDS3610 dataset. (A) Accuracy. (B) AUROC.

**Fig 6:**
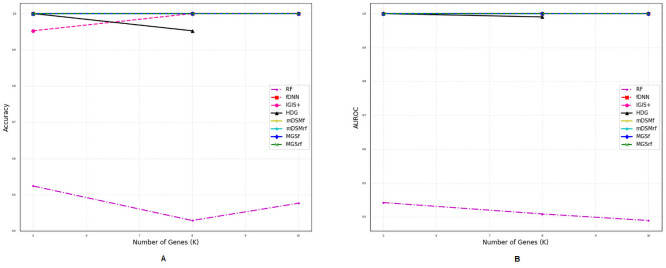
Performance comparison using different number of selected genes for the GDS4824 dataset. (A) Accuracy. (B) AUROC.

**Fig 7:**
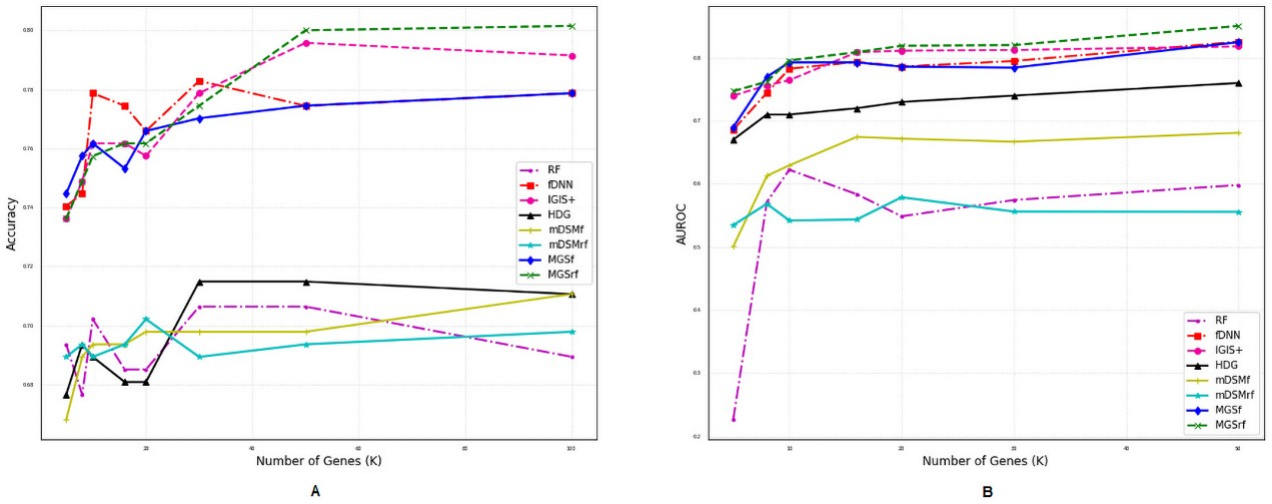
Performance comparison using different number of selected genes for the GSE106291 dataset. (A) Accuracy. (B) AUROC.

We also investigated the performances of the aforementioned methods for a different number of selected genes (*η*) using two metrics *accuracy* and *AUROC* as shown in Figs 2–7. Except *RF*, all the methods performed well (Figs 2–7).

In the case of balanced dataset (Figs 2 and 3), with GDS5306 and GDS4431 dataset, *MGS*_*f*_ and *MGS*_*rf*_ outperformed other methods which indicate that the proposed gene selection methods were able to select those genes which give additional information about the disease. For the small and highly imbalanced dataset GDS3610, our methods showed superior performances with different number of genes (Fig 5). With GDS3341 and GDS4824 datasets, all the gene selection methods classified the samples for different number of genes almost perfectly as shown in Figs 4 and 6. For these two datasets, the expression values of genes are more distinguishable between classes which would be the reason for the almost equal performance of every method. This might be the reason why the performance did not vary with an increase in the number of selected genes. We have also shown the strength of our methods with the GSE106291 dataset, which has a comparatively large number of samples (Fig 7).

Based on the results presented in Figs 2–7 and Tables 3 and 4, it is evident that the performances of *MGS*_*f*_ and *MGS*_*rf*_ are clearly better than the existing methods for balanced and imbalanced datasets. *MGS* performed well for all classifiers and thus, it is classifier independent. The datasets used for experimentation had a highly imbalanced distribution of the classes. This indicates that *MGS* is tolerant to imbalanced datasets. However, *MGS*_*rf*_ achieved slightly better performance for every value of *η*, indicating *MGS*_*rf*_ could classify more samples accurately than *MGS*_*f*_.

### Biological interpretation

It is not always a requisite that the selected genes with better classification ability are also relevant to a particular biological process. Therefore, to assess the performances of *MGS*_*f*_ and *MGS*_*rf*_, we investigated the ability of the top (≤ 10) selected genes to identify the most relevant pathways in the cancer types used in different balanced and imbalanced datasets (Tables 5 and 6).

**Table 5:** Comparative performance of different methods in identification of relevant biological pathways for balanced datasets.

Dataset ID	Cancer type	Method	No. of genes	Pathway	Output rank	FDR
GDS6063	Influenza A infected plasmacytoid dendritic cells(pDC)	*RF*	10	Cell cycle/HTLV-I infec./TGF-beta sig. path.	ND	-
		*fDNN*				
		*IGIS*+	1			
		*HDG*	8	Cell cycle	23	6.00E-01
				HTLV-I infection	16	2.39E-01
		*mDSM*_*f*_	2	Cell cycle	1	1.41E-06
				HTLV-I infection	2	7.63E-06
				TGF-beta signaling pathway	3	2.18E-05
		*mDSM*_*rf*_	2	Cell cycle	1	1.41E-06
				HTLV-I infection	2	7.63E-06
				TGF-beta signaling pathway	3	2.18E-05
		*MGS*_*f*_	2	Cell cycle	**1**	**1.41E-06**
				HTLV-I infection	**2**	**7.63E-06**
				TGF-beta signaling pathway	**3**	**2.18E-05**
		*MGS*_*rf*_	2	Cell cycle	**1**	**1.41E-06**
				HTLV-I infection	**2**	**7.63E-06**
				TGF-beta signaling pathway	**3**	**2.18E-05**
GDS5306	Breast cancer brain metastasis specimens and nonmetastatic primary breast tumors	*RF*	10	Cell cycle/Path. in cancer/TGF-beta sig. path	ND	-
		*fDNN*	10	Cell cycle	ND	-
				Pathways in cancer	6	1.96E-08
				TGF-beta signaling pathway	ND	-
		*IGIS*+	10	Cell cycle	ND	-
				Pathways in cancer	1	9.97E-08
				TGF-beta signaling pathway	ND	-
		*HDG*	4	Cell cycle	10	0.533
				Pathways in cancer	9	0.526
				TGF-beta signaling pathway	4	0.0525
		*mDSM*_*f*_	2	Cell cycle	2	3.64E-10
				Pathways in cancer	1	3.64E-10
				TGF-beta signaling pathway	3	1.71E-07
		*mDSM*_*rf*_	2	Cell cycle	2	3.64E-10
				Pathways in cancer	1	3.64E-10
				TGF-beta signaling pathway	3	1.71E-07
		*MGS*_*f*_	2	Cell cycle	**1**	**1.10E-12**
				Pathways in cancer	**2**	**3.92E-11**
				TGF-beta signaling pathway	**4**	**5.15E-08**
		*MGS*_*rf*_	2	Cell cycle	**1**	**1.10E-12**
				Pathways in cancer	**2**	**3.92E-11**
				TGF-beta signaling pathway	**4**	**5.15E-08**
GDS4431	Peripheral blood lymphocytes of autistic and non-autistic children	*RF*	10	Viral carcinogenesis	ND	-
				Hepatitis B	ND	-
				Ubiquitin mediated proteolysis	1	1.13E-04
		*fDNN*	10	Viral carcinogenesis	5	4.72E-04
				Hepatitis B	ND	-
				Ubiquitin mediated proteolysis	ND	-
		*IGIS*+	10	Viral carcinogenesis	2	6.72E-07
				Hepatitis B	ND	-
				Ubiquitin mediated proteolysis	ND	-
		*HDG*	10	Viral carcinogenesis	ND	-
				Hepatitis B	ND	-
				Ubiquitin mediated proteolysis	2	1
		*mDSM*_*f*_	10	Viral cycle/Hep. B/Ub. mediat. prote.	ND	-
		*mDSM*_*rf*_				
		*MGS*_*f*_	4	Viral carcinogenesis	**1**	**4.55E-03**
				Hepatitis B	**2**	**5.12E-03**
				Ubiquitin mediated proteolysis	**3**	**1.19E-02**
		*MGS*_*rf*_	4	Viral carcinogenesis	**1**	**4.55E-03**
				Hepatitis B	**2**	**5.12E-03**
				Ubiquitin mediated proteolysis	**3**	**1.19E-02**

ND—Not detected

FDR—False discovery rate

**Table 6:** Comparative performance of different methods in identification of relevant biological pathways for imbalanced datasets.

Dataset ID	Cancer type	Method	No. of genes	Pathway	Output rank	FDR
GDS3341	Nasopharyngeal carcinoma	*RF*	10	Viral carcinogenesis	ND	-
				Epstein-Barr virus infection	ND	-
		*fDNN*	10	Viral carcinogenesis	ND	-
				Epstein-Barr virus infection	ND	-
		*IGIS*+	3	Viral carcinogenesis	ND	-
				Epstein-Barr virus infection	ND	-
		*HDG*	10	Viral carcinogenesis	1	0.000121
				Epstein-Barr virus infection	ND	-
		*mDSM*_*f*_	4	Viral carcinogenesis	ND	-
				Epstein-Barr virus infection	ND	-
		*mDSM*_*rf*_	4	Viral carcinogenesis	ND	-
				Epstein-Barr virus infection	ND	-
		*MGS*_*f*_	10	Viral carcinogenesis	4	0.00259
				Epstein-Barr virus infection	14	0.166
		*MGS*_*rf*_	10	Viral carcinogenesis	**1**	**1.38E-14**
				Epstein-Barr virus infection	**4**	**2.56E-07**
GDS3610	Nasopharyngeal carcinoma	*RF*	10	Viral carcinogenesis	ND	-
				Epstein-Barr virus infection	29	0.53
		*fDNN*	10	Viral carcinogenesis	79	7.97E-08
				Epstein-Barr virus infection	113	6.85E-05
		*IGIS*+	7	Viral carcinogenesis	6	0.338
				Epstein-Barr virus infection	ND	-
		*HDG*	10	Viral carcinogenesis	10	3.82E-08
				Epstein-Barr virus infection	12	0.00000155
		*mDSM*_*f*_	9	Viral carcinogenesis	1	4.83E-13
				Epstein-Barr virus infection	5	0.0165
		*mDSM*_*rf*_	9	Viral carcinogenesis	1	4.83E-13
				Epstein-Barr virus infection	5	0.0165
		*MGS*_*f*_	10	Viral carcinogenesis	**1**	**4.83E-13**
				Epstein-Barr virus infection	**5**	**0.000259**
		*MGS*_*rf*_	10	Viral carcinogenesis	**1**	**4.83E-13**
				Epstein-Barr virus infection	**5**	**0.0165**
GDS4824	Prostate cancer	*RF*	10	ND	ND	-
		*fDNN*	10	Prostate cancer	28	0.435
		*IGIS*+	10	ND	ND	-
		*HDG*	8	Prostate cancer	7	0.632
		*mDSM*_*f*_	6	Prostate cancer	7	1.25E-16
		*mDSM*_*rf*_	6	Prostate cancer	7	1.25E-16
		*MGS*_*f*_	10	Prostate cancer	**6**	**1.29E-22**
		*MGS*_*rf*_	10	Prostate cancer	**6**	**1.29E-22**
GSE106291	Acute myeloid leukemia	*RF*	10	Chronic myeloid leukemia	ND	-
				Acute myeloid leukemia	ND	-
		*fDNN*	10	Chronic myeloid leukemia	ND	-
				Acute myeloid leukemia	ND	-
		*IGIS*+	10	Chronic myeloid leukemia	22	0.000319
				Acute myeloid leukemia	ND	-
		*HDG*	10	Chronic myeloid leukemia	ND	-
				Acute myeloid leukemia	ND	-
		*mDSM*_*f*_	10	Chronic myeloid leukemia	ND	-
				Acute myeloid leukemia	ND	-
		*mDSM*_*rf*_	10	Chronic myeloid leukemia	26	0.448
				Acute myeloid leukemia	ND	-
		*MGS*_*f*_	10	Chronic myeloid leukemia	ND	-
				Acute myeloid leukemia	ND	-
		*MGS*_*rf*_	10	Chronic myeloid leukemia	**1**	**2.78E-12**
				Acute myeloid leukemia	**8**	**8.74E-08**

ND—Not detected

FDR—False discovery rate

For balanced dataset, as this study primarily focused on disease data classification and the identification of relevant genes, we investigated the performances of all methods for capturing biological significance on various disease datasets derived from varied biological sources, such as cancer metastasis (GDS5306), autism (GDS4431) and viral infection (GDS6063). The results are shown in Table 5. GDS6063 dataset incorporates gene expression profiles of primary plasmacytoid dendritic cells following exposure to influenza A for 8 hours [50]. Human dendritic cells (DCs) are susceptible to infection with various viruses, including human T-lymphotropic virus Type 1 (HTLV-1), human immunodeficiency virus type 1 (HIV-1), measles virus and influenza virus [51]. In fact, the HTLV-1 acts more like the influenza virus in terms of infection to the DC cells and differs significantly with measles virus or HIV-1 [51]. The influenza virus-infected DCs induce a considerably higher proliferative response [51]. Transforming growth factor-beta (TGF-*β*) is a multifunctional cytokine and its activity increases during influenza virus [52, 53]. Along with *MGS*, *mDSM*_*f*_ and *mDSM*_*rf*_ could identify these as the top pathways (Table 5). Besides, *HDG* identified two pathways but other three methods could not identify any of the pathways. TGF-*β* signaling also plays an important role by stimulating cell invasion during metastasis of breast cancer [54, 55]. GDS5306 dataset contains gene expression data of HER2+ breast cancer brain metastasis specimens and HER2+ nonmetastatic primary breast tumors [50]. As shown in Table 5, *MGS*_*f*_ and *MGS*_*rf*_ identified pathways relevant to cancer and metastasis. Compared to *MGS*, *mDSM* and *HDG* performed reasonably well. GDS4431 dataset includes gene expression data of peripheral blood lymphocytes from autistic and non-autistic children [50]. Interestingly, the pathways indentified by majority of the methods overrepresented the pathways associated with cancer. It was recently reported that autism and cancer share risk genes [56]. Mutations in genes encoding the ubiquitin proteasome system (UPS) are associated with an increased risk for the development of autism spectrum disorders [57–59]. Viral carcinogenesis and ubiquitin mediated proteolysis were identified as the top pathways affected in autistic children. Connection between autism and Hepatitis B infection (one of the other top-ranked pathways) is not obvious based on the available information and may be explored in further studies.

For imbalanced datasets, it is evident that *MGS*_*f*_ and *MGS*_*rf*_ performed better in capturing the genes more relevant to the cancer type (Table 6). For example, Epstein-Barr virus (EBV) is well known to cause nasopharyngeal carcinoma (NPC), which is a type of epithelial cancer prevalent in Southeast Asia [60–62]. GDS3341 and GDS3610 datasets contain NPC samples [32, 33]. Although GDS3341 and GDS3610 are independent datasets, both *MGS*_*rf*_ and *MGS*_*f*_ could detect the genes involved in viral carcinogenesis and Epstein-Barr virus infection (Table 6). We used two different datasets (GDS3341 and GDS3610) on the same cancer type as built-in controls in the study to increase confidence with the experimental results. With both the datasets, *MGS*_*rf*_ and *MGS*_*f*_ performed almost equally well, although the genes selected by *MGS*_*rf*_ performed somewhat better. The other methods (*RF*, *fDNN*, *IGIS*+, *mDSM*_*f*_ and *mDSM*_*rf*_) could detect these pathways only for the GDS3610 dataset whereas *HDG* could detect pathways for both GDS3341 and GDS3610 datasets. In fact, *RF* and *IGIS*+ could detect one of these pathways. The GDS4824 dataset contains gene expression data from prostate cancer samples. Both the *MGS*_*rf*_ and *MGS*_*f*_ detected genes that are involved in prostate cancer. Although the prostate cancer pathway was ranked 6^th^ in the detected pathways (based on the FDR values) with the genes selected by the *MGS*_*rf*_ and *MGS*_*f*_, the top ranked pathways (FoxO signaling pathway, colorectal cancer, pancreatic cancer and endometrial cancer) are relevant to cancer as well [63–66]. In fact, unlike nasopharyngeal carcinoma, prostate cancer development involves different pathways. Fork head box O transcription factors (FoxO) regulates multiple cellular processes, including cell cycle arrest, cell death, DNA damage repair, stress resistance, and metabolism [67]. Inactivation of FoxO protein is linked to multiple tumorigenesis including prostate cancer [67–69]. Among the other methods, *fDNN*, *HDG* and *mDSM* could detect the genes associated with prostate cancer, although the rank of the pathway and associated FDR values were less significant.

**Fig 8:**
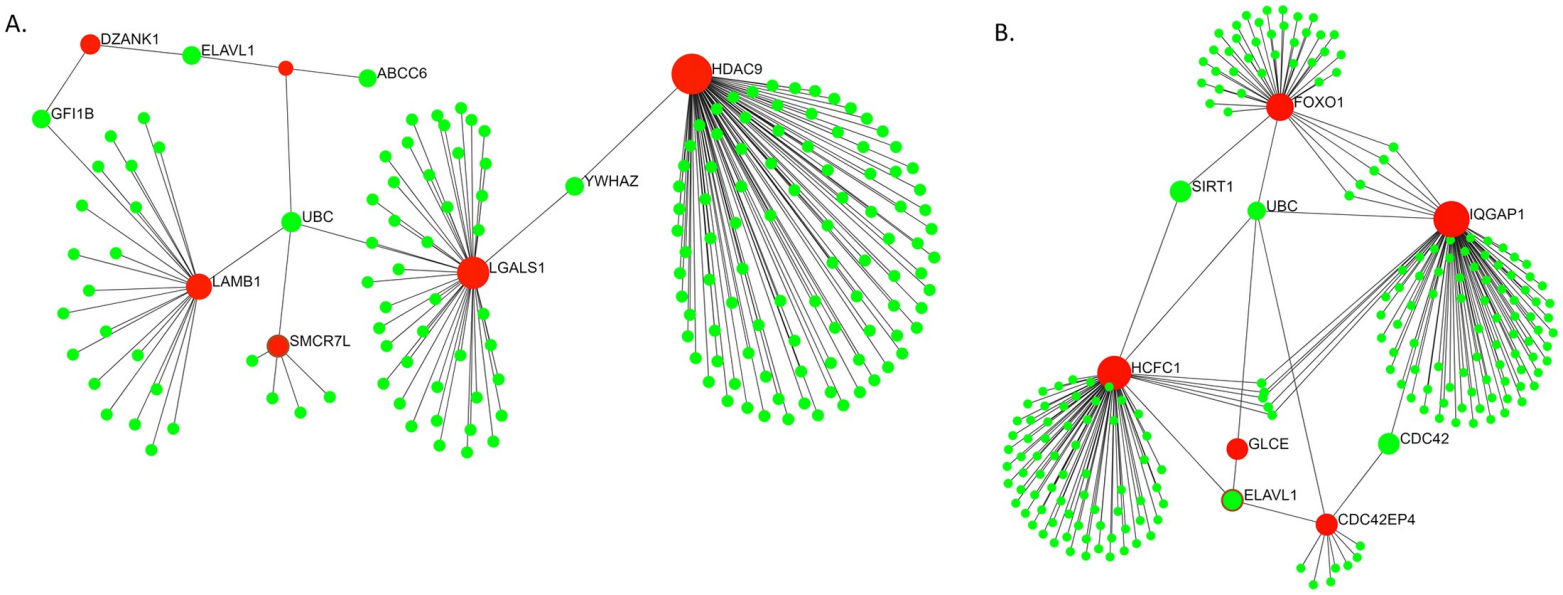
Roles of *MGS*_*rf*_ selected top genes in pathways related to cancer. (A) *LGALS*1 and *LAMB*1 were selected among the top 10 genes from GDS3341 dataset by the *MGS*_*rf*_. These (highlighted in red) are part of a sub-network that contains many other proteins (highlighted in green) known to play roles in different cancers [44]. (B) *HCFC*1, *FOXO*1 and *IQGAP*1 were selected among the top 10 genes from GDS4824 dataset by the *MGS*_*rf*_. These (highlighted in red) are part of a sub-network that contains many other proteins (highlighted in green) known to play roles in different cancers [44].

Although multiple proteins interact in a network inside a cell to attain a particular function, each of these does not play equally important role. Some proteins in a network are more connected and play a pivotal role in the overall biological process. *MGS*_*rf*_ and *MGS*_*f*_ selected top genes play important roles in pathways relevant to cancer (Fig 8).

It is noteworthy to mention that the proposed methods (*MGS*_*f*_ and *MGS*_*rf*_) performed better compared to the *mDSM* (*mDSM*_*f*_ and *mDSM*_*rf*_) despite sharing a closely similar methodology. These methods differed in the exclusion of redundancy term. *mDSM* discards a gene if it finds another gene with similar expression level. But as mentioned earlier, both genes may be informative despite redundancy and may provide useful information. Avoidance of the redundant genes may not be appropriate as genes working together in a pathway may be regulated in a more coordinated fashion than a random set of genes, and thus share a more coherent expression profile [70]. To understand this issue, let us consider an example of two genes named *MAN*1*C*1 and *ARCN*1 in dataset GDS3610. mDSM discarded *ARCN*1 gene since the redundancy value (0.685461) with *MAN*1*C*1 is greater than *χ*^2^ critical value (0.558168). Both of these genes work in pathways that inhibit cancer cell proliferation [71, 72]. Therefore, we did not consider redundancy in Eq 2 to select genes with *MGS*_*f*_ and *MGS*_*rf*_. Our proposed methods selected both *MAN*1*C*1 and *ARCN*1 as these provide additional information (0.598510).

## Conclusion

Here, we present a gene selection method followed by two gene ranking methods for the selection of informative genes from high dimensional low sample size gene expression data. The proposed gene selection method utilizes the maximum relevance and complementary information for selecting informative genes that have biological importance. Experimental results with known disease datasets illustrate that the proposed methods consistently achieve higher classification accuracy and select more biologically relevant genes than the previously reported methods. Moreover, we anticipate that the proposed method will also identify genes responsible for an unknown disease because it identifies effective and responsible genes for known diseases. However, there are a few challenges that need to be addressed in further studies. First, we believe that introducing a higher-order gene interaction may help to reduce the number of selected genes but it may increase the computational complexity. Second, a semi-definite programming based search strategy may help to obtain globally optimum gene subsets.

## Supporting information

S1 File (ZIP)
